# High acceptance of home-based HIV counseling and testing in an urban community setting in Uganda

**DOI:** 10.1186/1471-2458-11-730

**Published:** 2011-09-26

**Authors:** Juliet N Sekandi, Hassard Sempeera, Justin List, Micheal Angel Mugerwa, Stephen Asiimwe, Xiaoping Yin, Christopher C Whalen

**Affiliations:** 1 College of Public Health, University of Georgia, Athens, GA, USA; 2 School of Public Health, College of Health Science, Makerere University, Kampala, Uganda; 3 Yale Primary Care Program, Yale University School of Medicine, New Haven, CT, USA; 4 Uganda Case Western Research Collaboration, Kampala, Uganda

## Abstract

**Background:**

HIV testing is a key component of prevention and an entry point into HIV/AIDS treatment and care however, coverage and access to testing remains low in Uganda. Home-Based HIV Counseling and Testing (HBHCT) has potential to increase access and early identification of unknown HIV/AIDS disease. This study investigated the level of acceptance of Home-Based HIV Counseling and Testing (HBHCT), the HIV sero-prevalence and the factors associated with acceptance of HBHCT in an urban setting.

**Methods:**

A cross-sectional house-to-house survey was conducted in Rubaga division of Kampala from January-June 2009. Residents aged ≥ 15 years were interviewed and tested for HIV by trained nurse-counselors using the national standard guidelines. Acceptance of HBHCT was defined as consenting, taking the HIV test and receipt of results offered during the home visit. Multivariable logistic regression analysis was performed to determine significant factors associated with acceptance of HBHCT.

**Results:**

We enrolled 588 participants, 408 (69%, 95% CI: 66%-73%) accepted testing. After adjusting for confounding, being male (adj. OR 1.65; 95%CI 1.03, 2.73), age 25-34 (adj. OR 0.63; 95% CI 0.40, 0.94) and ≥35 years (adj. OR 0.30; 95%CI 0.17, 0.56), being previously married (adj. OR 3.22; 95%CI 1.49, 6.98) and previous HIV testing (adj. OR 0.50; 95%CI 0.30, 0.74) were significantly associated with HBHCT acceptance. Of 408 who took the test, 30 (7.4%, 95% CI: 4.8%- 9.9%) previously unknown HIV positive individuals were identified and linked to HIV care.

**Conclusions:**

Acceptance of home-based counseling and testing was relatively high in this urban setting. This strategy provided access to HIV testing for previously untested and unknown HIV-infected individuals in the community. Age, sex, marital status and previous HIV test history are important factors that may be considered when designing programs for home-based HIV testing in urban settings in Uganda.

## Background

Worldwide, HIV counseling and testing (HCT) is a key intervention for HIV prevention and a critical entry point into life-sustaining treatment and care programmes [1-4]. As the scale-up to anti-retroviral treatment access continues to accelerate in Sub-Saharan Africa, innovative strategies to increase access to HCT services should be simultaneously implemented [5]. In the past decade, a variety of HIV counseling and testing approaches have been used, including provider-initiated testing and counseling as part of medical care, and client-initiated or voluntary testing and counseling (VCT) [6]. Empirical evidence has demonstrated the public health benefits of HIV testing such as reduction in risky sexual behaviour [7,8] and linking HIV infected individuals to HIV care, treatment and support [9,10]. In Africa, however, low access and limited reach of facility-based HIV testing services have been an impediments to global attempts to prevent HIV transmission and scale-up of HIV care and treatment at population level [11].

Home-Based HIV Counseling and Testing (HBHCT) has the potential to address the challenges of limited access to testing. It involves the use of mostly community health workers to provide door-to-door counseling and testing services to consenting members of the community [12]. HBHCT has been shown to increase uptake of HIV testing, improve access to testing, while simultaneously limiting the costs for travelling to health facilities and reducing the potential stigma [8,12,13]. Uganda relies heavily on the conventional facility-based HIV testing models in line with the national policy recommendations [14], but these approaches mostly reach patients visiting health facilities or individuals who seek voluntary HIV testing following known exposure to HIV infection [15,16].

Although HBHCT may be effective in increasing access of HIV testing, a recent systematic review suggested that there is limited evidence and experience to support widespread scale-up of implementation of this model in Sub-Saharan Africa [11]. Few published studies have evaluated the feasibility, acceptance and uptake of HBHCT in rural [8,9,17] and in mixed rural-urban populations in Uganda [18]. Urban community residents represent a unique risk subgroup for HIV as evidenced by patterns of higher HIV sero-prevalence in urban compared to rural settings [19,20]. This pilot study was done to evaluate further the acceptance of HBHCT, to estimate the HIV sero-prevalence among individuals tested at home, and to determine the factors associated with acceptance of HBHCT in an urban setting. The information generated will add to the body of knowledge towards community-based interventions for HIV testing in urban settings.

## Methods

### Study Setting and Population

A cross-sectional house-to-house survey was nested in an ongoing community cough survey conducted in Rubaga division of Kampala city from January-June 2009. The division has a population of about 250,000 people, with 50% adults 15 years or older. It is composed of 13 administrative units called parishes which are further subdivided into 127 smaller units called local council villages or zones [21]. The division is mainly served by two private not-for-profit hospitals, two public health care clinics and is also proximal to the national referral hospital. All these health facilities offer provider-initiated HIV testing as part of routine medical care routine. However, HIV testing services are also available for a minimal cost at other stand-alone voluntary HIV counseling and testing centers in Kampala. Almost all participants resided no more than approximately 5-10 kilometers from at least one of the health facilities.

### Eligibility Criteria

Adults aged 15 years and older who were residents of Rubaga division were eligible to enrol in the study. We excluded persons who already knew their HIV status to be positive, those who were not able to communicate in English or Luganda, who did not give written consent to participate or who were absent from home at the time of survey. The study was approved by the Makerere University School of Public Health Research and Ethics committee and the Uganda National Council for Science and Technology.

### Sampling

Rubaga division was conveniently selected for the study because of its proximal location (~10 Km) to the Mulago National Referral Hospital which offers free comprehensive HIV/AIDS testing and treatment services. A computer-based random number generator was used randomly select five villages for the study. The total numbers of subjects to enroll per village were estimated using the proportion-to-population size approach. The specific houses within the selected villages were also selected randomly using an approach that was previously employed in a similar setting and described in detail elsewhere [22]. Eligible participants who were found at home were consecutively enrolled from the selected households at the time of the study visit. The five villages included in the study were similar in their population characteristics.

### Study Outcomes

The main study outcome was acceptance of HIV test which was operationalized as consenting, actually taking the test and receiving test results offered in the participants' home. The secondary outcome was the HIV sero-prevalence defined as participants who had a positive rapid HIV test result out of those who had a test performed.

### Data Collection

During the home visits a trained study team of six; three nurse counselors and three interviewers worked in pairs to obtain written informed consent, administered face-to-face interviews and, performed HIV testing. The information gathered included socio-demographics, occupation, weekly income, previous HIV test history, knowledge if current HIV status and, willingness to accept an HIV test. Quality assurance principles of counseling were observed before, during and after the HIV test in accordance with the national HV policy guidelines [14]. Eligible members of the same household were offered the options of individual or group pre-test counseling. For household members who preferred group counseling, the study counselors provided the standard HIV information about testing in one seating after which they were requested to give written consent to be tested. Post-test counseling and delivery of results was offered according to the participants' preference.

### Home-Based HIV Testing Procedures

Rapid HIV testing was performed according to the testing algorithm recommended by the Uganda national HIV testing policy [14]. Determine HIV-1/2 assay (Abbott Laboratories, Illinois, United States of America) was used for screening, the HIV-1/2 STAT-PAK Dipstick assay (Chembio Diagnostic System Inc, New York, USA) was used as the confirmatory test and the Uni-Gold test (Trinity Biotech, Wicklow, Ireland) as the tie-breaker. The results were reported as positive if the Uni-gold test results were positive. The average test result turnaround was 15-20 minutes.

### Referral for Care and Medical Evaluation

Participants who were identified to be HIV sero-positive during the survey were advised to seek further evaluation and referred to HIV care centres of their preference. Individuals who tested HIV negative received HIV prevention counseling. The interviewers also encouraged participants to disclose their HIV status to their sexual partners and/or family members as they felt comfortable.

### Statistical Analysis

The main outcome was coded as a binary variable (HIV test acceptance, 'Yes' or 'No'). Acceptance was expressed as the proportion (with 95% confidence intervals) of participants who voluntarily accepted and took the HIV test among those who were offered a test. The HIV sero-prevalence was estimated among those who accepted to be tested from the HBHCT as the proportion who were HIV sero-positive. Differences in proportions among groups for the unordered categorical variables were tested with Pearson's chi-square; trends in proportions of ordered categorical variables were tested for using the Cochran-Armitage test. Multivariable logistic regression analysis was used to determine factors that were associated with acceptance of HBHCT. The best prediction model was selected based on a measure of model goodness of fit, the Akaike Information Criterion (AIC) [23]. Factors included in the analysis were sex, age, religion, education, marital status and previous HIV testing. All chi-square P-values ≤ 0.05 were considered statistically significant. Data management and analysis was done using Statistical Analysis Software (SAS) and cross-validated using STATA (version 11.0; StataCorp, LLC, College Station, TX, USA).

## Results

**Figure 1:**
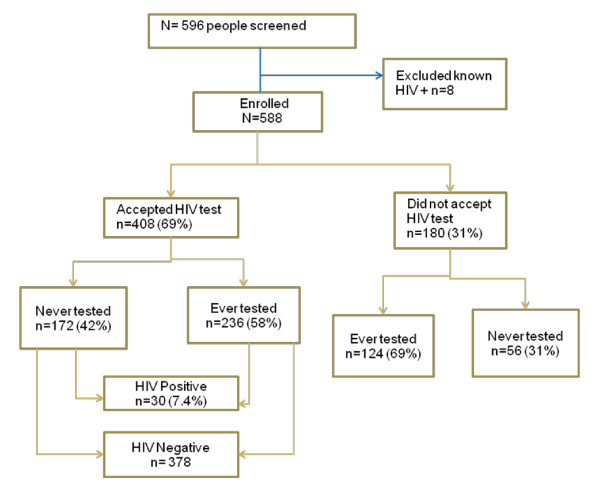
**Study Flow Chart**.

Between January and June, 2009, 444 households comprising 698 potential participants were surveyed by the study counselor-interviewer teams. Of the 698 individuals, 588 (84%) were successfully enrolled on the study (Figure 1). Individuals who did not respond (n = 110; 16%) were not at home at the time of the survey or declined to participate. There was 100% response rate on all items in the questionnaire. All participants who accepted to take the HIV test underwent post-test counseling and received their test results.

### Description of Study Participants

Participation was skewed towards women as only 22% of the sample was men. The median age was 23 years (IQR = 10), with the majority (58%) in the age group 15-24 years [Table 1]. Nearly half (48%) of the participants were currently married and the majority identified themselves as Christians. More than half (53%) had attained at least 7-13 years of formal education) and had a relatively low average weekly income of not more than five thousand Uganda shillings (~2.00 USD). About two-thirds of the of the study participants self reported as ever had an HIV test, with the majority reporting having had a test within the past twelve months. However, 39% of the participants had never tested for HIV; this roughly reflects the unmet need for HIV testing at that time in this community.

**Table 1:** Baseline Characteristics of Participants in Home-Based HIV Testing in Rubaga, Kampala (January - June 2009)

Characteristics	Frequency N = 588	Percent
Age, yrs*		
15-24	337	58
25-34	167	28
≥ 35	84	14
**Sex**		
Female	456	78
Male	132	22
**Marital status**		
Never married	235	40
Currently married	281	48
Previously married	72	12
**Religion**		
Catholic	161	27
Protestant	161	27
Muslim	141	24
Others**	125	22
**Education level**		
None-Primary	221	38
Secondary	312	53
College/University	55	9
**Weekly Income†**		
None	145	25
> 0-5000 (Ugshs)	237	40
≥ 5000	206	35
**Ever had an HIV test**		
Yes	360	61
≤ 12 months	216	37
> 12 months	144	26

* Mean (SD) = 26 (8.9), Median Age (IQR) = 23 (19, 29)

** Other -(Pentecostal, Seventh day Adventist or religion not specified)

**† **Median weekly income = UGSH 4,000 (~ US$2.00)

### Acceptance of Home-Based HIV testing

Among participants enrolled in the study, 408 (69%, 95% CI: 66%-73%) accepted home-based HIV counseling and testing. The distribution of baseline characteristics for participants who accepted and those who did not accept differed by age (p = 0.012), marital status (p = 0.017), education level (p = 0.043) and previous HIV test history (p ≤ 0.0001). The proportion of participants who accepted an HIV test in the home was lower for those who had never been tested previously compared with those who had tested in the past (58% vs. 85%). The two groups were similar in sex and religion distribution (p = 0.112 and 0.171) [Table 2].

**Table 2:** Bivariate Analysis of Factors Associated with Acceptance of HIV Testing in Kampala, Uganda N = 588

Characteristics	Accepted HIV testing N = 408 (69%) n (%)	Did not Accept HIV testing N = 180 n (%)	Pearson X^2 ^P-value	Unadjusted OR(95% CI)
**Sex**				
Female	309(76)	147 (82)		1.00
Male	99 (24)	33 (18)	0.112	1.43(0.92-2.22)
**Age, yrs**				
15-24	245(60)	92 (51)		1.00
25-34	114(28)	53(29)		0.81 (0.54-1.21)
> = 35	49(12)	35(20)	0.012*†	**0.53 (0.32-0.86)**
**Marital status**				
Never Married	169(41)	66(37)		1.00
currently	181(44)	100(56)		0.71 (0.49-1.03)
Previously	58(15)	14(7)	0.017*	1.62 (0.85-3.00)
**Religion**				
Catholic	112 (27)	49(27)		1.00
Protestant	109(26)	52(39)		0.92 (0.57-1.47)
Muslim	91(23)	50(28)		0.80 (0.49-1.29)
Other	96(24)	29(16)	0.171	1.45 (0.85-2.47)
**Education level (yrs of school)**				
None - Primary	164(40)	57(32)		1.00
Secondary	209(51)	103(57)		0.71 (0.48-1.03)
College/University	35(9)	20(11)	0.043*†	0.61(0.33-1.14)
**Ever had HIV test**				
No	172 (42)	56(31)		1.00
Yes				
≤ 12 months	125(31)	91(51)		**0.45 (0.30-0.67)**
> 12 months	111(27)	33 (18)	< 0.0001*	1.10(0.067-1.79)

**$ Other **-(Pentecostal, Seventh day Adventist or unspecified religion)

*significant at P ≤ 0.05 **† **trend P value (Cochran-Armitage)

### Factors Associated with Home-based HIV test Acceptance

In a multivariable logistic regression analysis, sex, age, marital status and previous HIV test history were significantly associated with acceptance of HIV testing [Table 3]. Males were nearly twice as likely to accept the HIV test compared to females [adjusted OR: 1.67, 95% CI: 1.03-2.73]. Participants in age groups 25-34 and 35 years or older were less likely to accept the HIV test as compared with younger adults aged 15-24 years. Participants who were previously married were more than three times more likely to accept the HIV test when compared with those who were never married. People who reported to have previously tested for HIV within 12 months were less likely to accept the HIV test when compared with those who had never tested. Those who had tested more than 12 months prior were more likely to accept the test compared to those who had never tested although the association was not statistically significant.

**Table 3:** Multivariable Regression Analysis of Factors Associated with Acceptance of HIV Testing in Kampala, Uganda N = 588

Characteristics	Accepted HIV testing N = 408 n (%)	Adjusted OR (95% CI)
**Sex**		
Female	309(76)	1.00
Male	99(24)	**1.67(1.03-2.73)**
**Age, yrs**		
15-24	245(60)	1.00
25-34	114(28)	**0.63 (0.40-0.94)**
> = 35	49(12)	**0.30 (0.17-0.56)**
**Marital status**		
Never married	169(41)	1.00
Currently	181(44)	1.25 (0.75-1.93)
Previously	58(15)	**3.22 (1.49-6.98)**
**Ever had HIV test**		
No	172 (42)	1.00
Yes		
≤ 12 months	125 (31)	**0.50 (0.30-0.74)**
> 12 months	111 (27)	1.28 (0.75-2.19)

*significant at at P ≤ 0.05

### HIV Sero-Prevalence among Participants who Accepted Home-Based HIV Testing

Home-based HIV testing identified 30 [7.4%, 95% CI: 4.8%- 9.9%] previously undetected HIV cases among the 408 participants who accepted the test. However the estimated HIV sero-prevalence was 6.5% (95% CI: 4.5-8.5%] for the entire study sample including eight people who were already known to be HIV positive. The majority of those who were found to be HIV sero-positive were young adults 15-24 years, female, married, of very low education level [Table 4]. Most had "never tested" for HIV in the past, though nearly one-third of the newly detected HIV sero-positive individuals had taken an HIV test within 12 months prior of the home-based testing survey. There were no significant differences in baseline characteristics between participants that tested HIV sero-positive and those that tested sero-negative except for marital status. The most predominant reason for not accepting home-based testing was having taken a test within 6 months prior to the survey, (33.3%). Other reasons included fear or not being emotionally ready to take an HIV test (19.4%), wanting to first seek approval of a spouse or parent (9.4%), not being interested in taking a test (18.9%) or not being sexually active (7.2%), these numbers are not presented in the table.

**Table 4:** HIV Sero-Prevalence Among 408 Adults Who Accepted Home-based Testing in Kampala: January - June, 2009

Characteristic	HIV positive n = 30 (7.4%)	HIV negative n = 378 (%)	Chi-sq P
**Age, yrs**			
15-24	16(53)	229 (61)	
25-34	8 (27)	106 (28)	
≥ 35	6 (20)	43 (11)	0.370
**Sex**			
Female	25(83)	284 (75)	
Male	5(17)	94 (25)	0.313
**Marital Status**			
Never Married	6 (20)	163(43)	
Previously Married	8 (27)	50(13)	
Married	16(53)	165(44)	**0.021**
**Religion**			
Catholic	11(37)	101(27)	
Protestant	7(23)	102 (27)	
Muslim	6(20)	85 (22)	
Other	6(20)	90 (24)	0.708
**Education level (yrs of school)**			
None - Primary	17 (57)	147 (39)	
Secondary	11 (37)	198 (52)	
College/University	02 (6)	33(9)	0.160
**Ever had HIV Test**			
Never tested	13 (43)	159 (42)	
≤ 12 months	9 (30)	116 (31)	
> 12 months	8 (27)	103 (27)	0.268

## Discussion

We found that home-based HIV counseling and testing was feasible and acceptable (69%) in this urban setting in Kampala. The factors associated with acceptance of HIV counseling and testing in the home were being 25 years or older, being male, previously married and previous HIV testing history. The HIV sero-prevalence in the study population was similar to the most recent national HIV sero-prevalence estimate of 6.4% [24]. These findings were consistent with population-based studies done elsewhere that showed high acceptance for home-based HIV counseling and testing [3,25]. However, we did not find any published quantitative urban population-based studies for Uganda.

Our study findings support the use of a home-based HIV counseling and testing strategy in an urban setting. Urban population-based studies done in Zambia and Kenya reported similar acceptance levels; 71% and 78% of participants agreed to HIV testing and counseling in the home, respectively [3,25]. In contrast, rural population-based studies done in Africa consistently showed higher levels of acceptance for home-based HIV counseling and testing ranging from 84% to 98% [12,17,26]. The urban-rural differences in acceptance for home-based HIV counseling and testing could be due to underlying disparities in availability and access to testing sites. Regardless of the contextual factors, the high demand for home-based HIV testing suggests that this strategy could potentially address known barriers associated with facility-based HIV testing including stigma, discrimination, fear to receive results, lack of confidentiality, privacy, long distances and cost of transportation to testing sites [5,9,13,16]. Based on the reasons given by those individuals for not accepting to take the home-based test in our study such as not being emotionally prepared and having to consult spouses or parents, it is evident that HIV programs would still need to be aware of existing barriers and seek to address them.

Like other studies [25], we found that men were more likely to accept the test than women during this home-based survey. Differences in gender r roles may explain some of the results in this study. Women of reproductive age are more likely than men to come into frequent contact with the health system and thus to access HIV testing services. For example, pregnant women have an opportunity to learn their HIV sero-status through the routine testing during Antenatal Care and Prevention of Mother-to-Child HIV transmission programs [19]. On the other hand, the higher acceptance of HBHCT by men presents an opportunity to reach entire families through community-based approaches. From a social-cultural perspective, targeting married men with home-based HIV counseling and testing could be beneficial for promoting couple counseling and testing [10]. In Uganda, men often play a dominant role in the decision making process related to couple and family health issues.

Consistent with a similar study done in Malawi [27], socio-demographic factors also influence acceptance of home-based HIV testing, older individuals were less likely to accept a home-based HIV test compared to younger individuals. A possible explanation is that older individuals are usually married or in stable relationships and may have a lower HIV risk perception. However, previously married (widowed or separated) individuals were more likely to accept the HIV test; perhaps motivated by a higher risk perception for HIV infection in this subgroup. In Uganda, being widowed often results from death of spouse from HIV/AIDS [27] and has been shown to be a risk factor for HIV infection [20]. Previous testing especially within 12 months prior to the HBHCT was negatively associated with acceptance compared with individuals never previously tested. The Uganda HIV testing policy recommends repeating an HIV testing within six months of a previous test [14] but there is need to be a more practical strategy for its implementation.

The HIV sero-prevalence in the overall study population was 6.5% while the sero-prevalence among those who accepted to test was 7.4%. These findings are very similar to the national sero-prevalence of 6.4% and provides external validity to our study [28]. A lower proportion of individuals self-reported as previously "never tested" (39%) compared to 55%-65% national estimates for 15-49 year old urban residents [19]. Furthermore, a high proportion of previously "never tested" individuals were HIV sero-positive. Similarly, a study done in rural western Uganda was effective in identifying previously unknown HIV positive individuals as well as discordant couples among those who had "never tested" [17]. Since Uganda's HIV epidemic has reached a stable and generalized state, it is critical to identify the hidden drivers for effective infection prevention and control. This study and others [1,9,17] point to the potential value of home-based HIV counseling and testing as a way to reach untested individuals.

The WHO/UNAIDS policy guidelines recommends four key strategies for HIV counseling and testing that are mainly facility-based [16]. However, it is apparent that complementary strategies such as home based HIV counseling and testing that expand access to HIV testing services in underserved or hard to reach communities are needed. Such strategies could enhance early identification of HIV positive individuals, prompt linkage to treatment and care, and may curtail risky sexual behaviours that lead to HIV transmission [29]. The successful Implementation of HBHCT could be hampered by shortage of trained health workers and high operational costs in resource limited settings. In order to circumvent such challenges, delivery models that leverage community resources such as trained community lay providers who can perform rapid HIV testing under supervision by medical personnel, have been used elsewhere [12] and are also endorsed by the Uganda HIV policy guidelines [14]. Integration of HBHCT with other community screening programs such as those for tuberculosis and/or malaria may also increase cost-effectiveness [13].

### Study Limitations

Our study findings should be interpreted in light of some limitations. First, the sero-prevalence of the study could be an underestimate because we are missing information about the individuals who did not accept the HIV test. If we apply the estimated 6.4% sero-prevalence to our study sample then we would have expected a slightly higher HIV prevalence. We also excluded children younger than 15 years; therefore our findings can only be generalized to the adult population in similar urban settings. Second, there is selection bias as the study population comprises mostly women; this may lead to an overestimation of the HIV sero-prevalence because women are at a greater risk for HIV than men [20]. The strength of this study is that it was conducted in the general urban population which gives a picture of the operational experiences and challenges that could be encountered when HBHCT is undertaken in similar settings. Previous community HIV testing has been mainly targeted towards high risk household members of already known HIV/AIDS infected or tuberculosis index cases [10].

## Conclusions

Acceptance of home-based counseling and testing was relatively high in this urban setting. This strategy provided access to HIV testing for previously untested and unknown HIV-infected individuals in the community. Age, sex, marital status and previous HIV test history are important factors that may be considered when designing programs for home-based HIV testing in urban settings in Uganda.

## Competing interests

The authors declare that they have no competing interests.

## Authors' contributions

JNS: Designed the study, coordinated recruitment of participants, collected data, analyzed data and drafted the manuscript. CCW: Designed the study, participated in data analysis and reviewed all drafts of the manuscript. HS: Designed the study, participated in data collection, analysis, and reviewed all drafts of the manuscript. JL: Designed the study, coordinated recruitment of participants, collected data, analyzed data and reviewed drafts of the manuscript. AS: Participated in design, data analysis and reviewed all draft manuscript. MA: Participated in design, data collection, analysis, reviewed drafts of the manuscript. XY: Guided and help with data analysis, reviewed drafts of the manuscript. All authors read and approved the final manuscript.

## Pre-publication history

The pre-publication history for this paper can be accessed here:

http://www.biomedcentral.com/1471-2458/11/730/prepub
